# Multifaceted impacts of nanoparticles on plant nutrient absorption and soil microbial communities

**DOI:** 10.3389/fpls.2024.1497006

**Published:** 2024-11-13

**Authors:** Hanfeng Zhang, Tiantian Zheng, Yue Wang, Ting Li, Qing Chi

**Affiliations:** ^1^ National Key Laboratory of Cotton Bio-breeding and Integrated Utilization, School of Agricultural Sciences, Zhengzhou University, Zhengzhou, China; ^2^ Henan Key Laboratory of Ion-Beam Green Agriculture Bioengineering, School of Agricultural Sciences, Zhengzhou University, Zhengzhou, China

**Keywords:** nanoparticles, plants, nutrient uptake, soil, rhizosphere microbial communities

## Abstract

With the growth of the global population and the increasing scarcity of resources, the sustainability and efficiency improvement of agricultural production have become urgent needs. The rapid development of nanotechnology provides new solutions to this challenge, especially the application of nanoparticles in agriculture, which is gradually demonstrating its unique advantages and broad prospects. Nonetheless, various nanoparticles can influence plant growth in diverse manners, often through distinct mechanisms of action. Beyond their direct effects on the plant itself, they frequently alter the physicochemical properties of the soil and modulate the structure of microbial communities in the rhizosphere. This review focuses intently on the diverse methods through which nanoparticles can modulate plant growth, delving deeply into the interactions between nanoparticles and plants, as well as nanoparticles with soil and microbial communities. The aim is to offer a comprehensive reference for the utilization of functionalized nanoparticles in the agricultural sector.

## 1 Introduction

In recent years, with the rapid development of agricultural technology and the increasing awareness of environmental protection, the demand for new materials and technologies in the field of agricultural production has become increasingly urgent (Moretti and Marucci, 2019; Magnabosco et al., 2023). The prolonged use of chemicals, pesticides, and fertilizers can indeed ease the challenges of food security in the long term. However, this practice poses risks such as contamination, soil fertility loss, non-target species impact, disease/insect resistance, biodiversity decline, and harm to humans/animals (Yousef et al., 2023; Chandrasekaran and Paramasivan, 2024). Consequently, there is an urgent need for innovative and efficient agricultural technologies to address the global challenges of food production and security (Thorakkattu et al., 2024).

Nanoparticles, often abbreviated as NPs, are regarded as materials ranging from 1 to 100 nm in size, and have different sizes, geometry, physical shape, mechanical strength and chemical composition, which have attracted people attention due to their wide application prospects (Lowry et al., 2019; Ravichandran et al., 2021; dos Santos et al., 2022; Garg et al., 2024; Sun et al., 2024). Nanotechnology has been widely applied across various sectors, including biomedicine, agriculture, and environmental remediation. The United Nations Food and Agriculture Organization (FAO) and the World Bank are actively encouraging the integration of nanotechnology into agricultural practices, with the development of sustainable agricultural systems being a central goal of current nanotechnological applications (De Chiffre et al., 2003; Mishra et al., 2017; Kah et al., 2019; Ahmad et al., 2022; Wang et al., 2022). Compared to traditional agricultural technology, nano-agricultural technology offers numerous advantages, closely linked to enhancements in production efficiency, reductions in input costs, and diminished ecotoxicity (Servin et al., 2015; Kah et al., 2019; Zhang et al., 2024). For example, zinc oxide, silver oxide nanoparticles had been explored as effective slow-release nanofertilizers, transport carriers, and bacteriostatic agents to provide plants with essential nutrients and inhibit pathogens, thus promoting plant growth and increasing crop yields (Elhaj Baddar and Unrine, 2018; Sun et al., 2018; Shireen Akhter Jahan et al., 2024). The application of nanoparticles to soil can influence its physical and chemical properties, the metabolic richness of plant roots, and the activity of the rhizosphere microbial community (Dimkpa et al., 2013; Sarma et al., 2024). Furthermore, the physical and chemical characteristics of soil, including texture, organic matter content, and pH level, inherently influence the migration and morphology of nanoparticles within the soil, which impact the bioavailability of nanoparticles (Cornelis et al., 2014; Reith and Cornelis, 2017; Gómez-Sagasti et al., 2019). The ecological functions of nanoparticles and their environmental impacts are current research focal points. However, their effects on soil health and agricultural applications are of greater practical significance. In particular, the influence of nanoparticles on rhizosphere microorganisms and plant physiology is crucial for enhancing research into sustainable agricultural development strategies.

Land plants interact with soil microorganisms through their roots, making rhizosphere microbial community crucial for soil health and crop growth (Ding et al., 2019; Zhou et al., 2020; Kusiak et al., 2022). Compared to the perpendicular soil (soil not connected to the roots and soil falling after root shaking) (Ding et al., 2019), rhizosphere soil (the soil within 1 mm of the root surface) is teeming with a multitude of microorganisms exhibiting high biological and chemical activity, which is directly linked to the stability and productivity of agricultural production systems (Kaye et al., 2005; Edwards et al., 2015). Numerous recent studies have demonstrated the enhanced efficacy of nanoagents in regulating the plant rhizosphere microbiome compared to traditional non-nanometer approaches (Ahmed et al., 2022; Ahmed et al., 2023). Moreover, the regulation of microbiomes using nanoagents has the potential to enhance plant growth through a variety of mechanisms (Ahmed et al., 2022; Ahmed et al., 2023). Secondly, nanoparticles can indirectly promote plant nutrient absorption and ultimately promote plant growth by increasing the richness of rhizosphere microbiota (Xu et al., 2023). For example, pepper plants treated with Nano-selenium could significantly enhance the presence of beneficial microorganisms in the rhizosphere soil, including *Gammaproteobacteria*, *Alphaproteobacteria*, *Bacteroidetes*, *Gemmatimonadetes*, and *Deltaproteobacteria*, as well as *Anaerolineae (* Li et al., 2022). These alterations in microbial communities lead to a substantial increase in soil enzyme content, soil metabolites such as fluorescein diacetate, urease, brassinosteroids, and p-hydroxybenzoic acid, and plant metabolites like rutin, luteolin, brassinosteroids, and abscisic acid, which enhanced the contribute to bolstering plant defense mechanisms and improving plant growth (Li et al., 2022). Rhizosphere microorganisms promoted plant growth by providing nutrients and hormones, while the metabolites secreted by plant roots could also change the species and number of rhizosphere microbial communities (Hwangbo et al., 2016; Khoiri et al., 2024). Consequently, the creation and application of suitable nanoparticles in agriculture are anticipated to not only improve but potentially supplant the use of chemical pesticides and fertilizers.

This review delves into the utilization of nanoparticles within agricultural production, examining how these particles can foster plant growth through various mechanisms: (i) enhancing nutrient absorption, (ii) facilitating controlled release of nutrients, (iii) enabling precise delivery of nutrients to targeted locations, and (iv) augmenting the population of beneficial microorganisms in the rhizosphere while suppressing those that are pathogenic and detrimental to plant development. Nanoparticles can stimulate plant growth both directly, by enhancing physiological processes, and indirectly, by modulating the beneficial microorganisms in the rhizosphere and altering soil conditions. Consequently, strategies driven by nanotechnology offer a promising and sustainable approach to boost crop growth and bolster crop resilience against stress factors.

## 2 Nanoparticles enhance nutrient absorption

**Figure 1:**
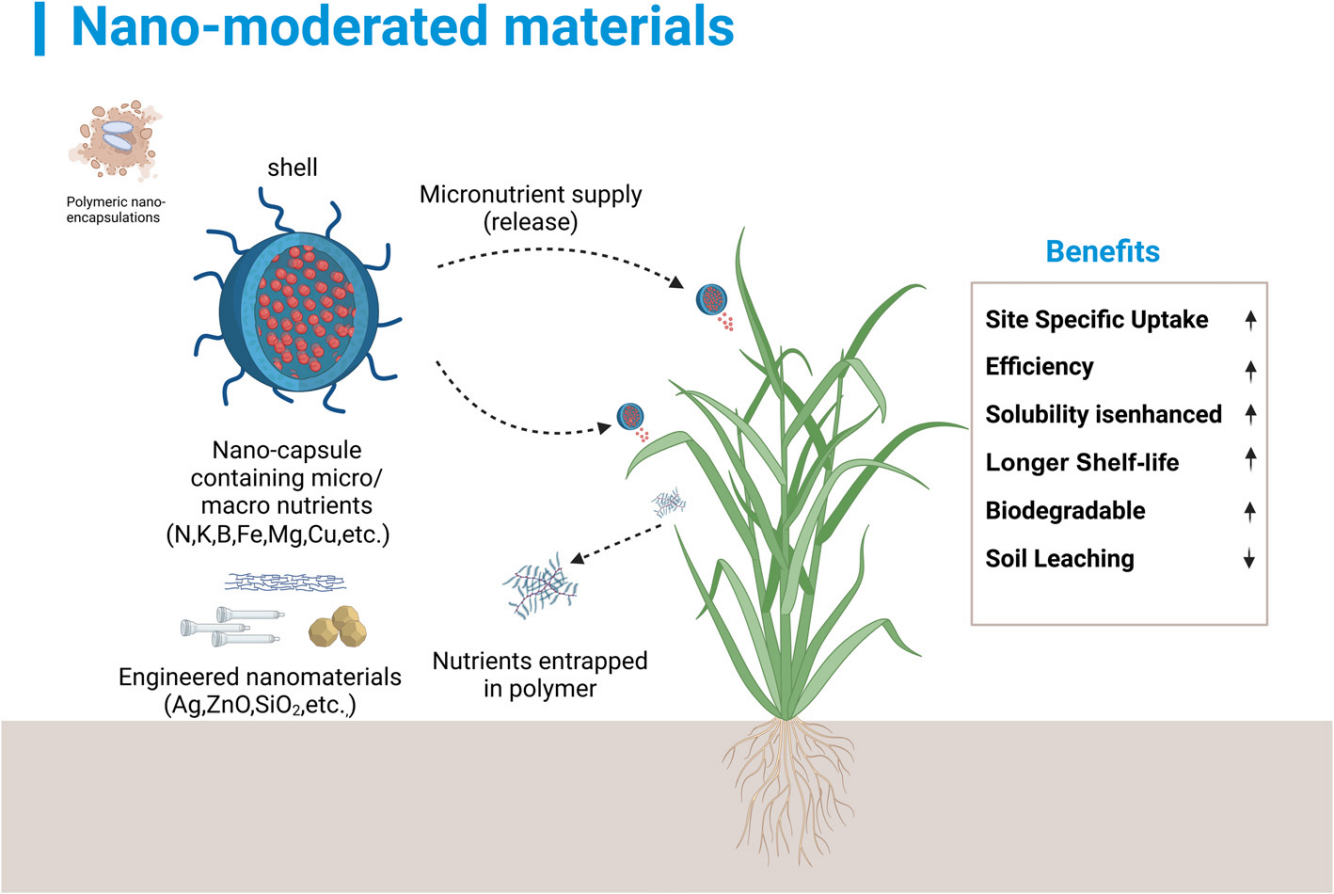
Nanoparticle-based protective agents or carriers are designed to regulate the discharge of active compounds, enhance stability, and control the release rate, thereby achieving sustainable agricultural practices.

Nanoparticles hold great potential for enhancing nutrient uptake, and certain nanoparticles can improve plant nutrient utilization efficiency by employing mechanisms such as directional delivery, sustained or controlled release (Solanki et al., 2015; Dutta et al., 2022; Taware et al., 2024) (**Figure 1**). During the promotion of plant nutrient uptake, nanoparticles can influence nutrient absorption in two ways. On the one hand, they can act as carriers for nutrients, and on the other, they can modulate soil microorganisms to enhance the plant’s nutrient absorption capabilities (Bortoletto-Santos et al., 2020; Khan et al., 2022); Conversely, nutrient elements could be precisely delivered to various regions of plants via nanoparticle carriers, thereby enhancing the efficiency of nutrient utilization (Fincheira et al., 2021).

### 2.1 Nanoparticles function as carriers for slow-release fertilizers, enhancing the absorption of nutrients by plant roots.

At present, nanoparticles find extensive application in the realms of energy, electronics, and architecture, yet their utilization in agriculture-related domains remains comparatively limited (de Silva et al., 2020). It possesses the potential for the slow release of fertilizers, owing to its diminutive size, substantial surface area, robust adsorption capacity, and the capability to control the release kinetics at the target site (Ghormade et al., 2011; Zulfiqar et al., 2019; Al-Mamun et al., 2021). The nanoparticles’ diminutive scale and elevated reactivity allow them to penetrate the plant cell wall with greater ease, thereby enhancing the transport and absorption of nutrients throughout the plant (Chhipa, 2017). Furthermore, it enhances fertilizer adsorption efficiency and stability, allowing slow, sustained nutrient release. This fosters plant growth, preserves beneficial microbiota diversity, mitigates eutrophication runoff, and prevents pollution (Kalwani et al., 2022).

Research conducted on nanofertilizers in aqueous environments revealed that a 40% urea-hydroxyapatite formulation demonstrated a controlled release of nitrogen, capable of sustaining the process for up to one week., while pure urea depleted within just a few minutes (Kottegoda et al., 2017). Likewise, the gradual and continuous release of urea from urea-silica nanohybrids prevents premature depletion of urea, ensuring effective and precise delivery of nitrogen and silica to the plant (de Silva et al., 2020).

Nanoparticles can also exert their slow-release function within soil media, markedly enhancing the efficiency of nutrient utilization. For instance, in the study of indigenous wheat irrigated nanohybrid, it was found that urea molecules were slowly released in doped Zn and Mg hydroxyapatite nanohybrids for up to two weeks, and this nanocompound containing only a 50% nitrogen dose maintained wheat crop yield and nitrogen nutrient uptake (Sharma et al., 2022). The recent research had revealed that a nanocarrier with a core and shell structure, composed of urea-loaded Metal-Organic Frameworks (MOFs) and silica, can facilitate the sustained release of nitrogen to crops. When utilizing urea/MIL-100(Fe)/silica heterojunction nanomaterials to treat potted rice, the nitrogen use efficiency of the rice was found to be 34.7% greater than that achieved with traditional urea treatment (Wu et al., 2024).

During the growth cycle of rice, urea-coated hydroxyapatite nanoparticles (urea coated hydroxyapatite nanoparticles, UHA) were released more slowly than conventional urea (Bhavani et al., 2020). Nano U-NPK(containing Ca, P, K, NO_3_ and urea multi-nutrient nanofertilizer) not only ensures the slow release of the most crucial plant macronutrients(N, P, K), but also has the potential to reduce the nitrogen supply to plants by 40% (Ramírez-Rodríguez et al., 2020). Potassium-based nanoparticles (K_2_SiO_3_-NP, K_18_Mo_8_O_33_-NPs) compared with traditional potassium fertilizer, its utilization efficiency was about 40% higher in soybean growth, mainly due to the slow release effect of the nanoparticles, which can provide potassium ions for a longer time, effectively avoiding the toxic effect of large dose rapid delivery (Wang et al., 2024). The montmorillonite nano-hybrid composite was capable of decelerating the release rate of nitrogen across various pH conditions, extending its duration to foster plant growth (Madusanka et al., 2017). The latest study found that a special slow-release fertilizer: milk, could be made into high-fertilizer fertilizer rich in fulvic acid and potassium. The pot experiment results showed that the slow-release fertilizer was slow and more significant fertilizer effect than the traditional organic fertilizer and potassium fertilizer, and had good acid soil restoration effect (Zhu et al., 2024). In conclusion, tailored nanofertilizers have the potential to not only enhance plant growth but also to improve the soil’s physiochemical properties, playing a pivotal role in the advancement of sustainable agriculture.

### 2.2 Nanoparticles are capable of precisely delivering nutrients

Nanoparticles had improved the nutrient uptake in plants through the precise application of chemical fertilizers and plant growth regulators, thereby fostering innovation within the agricultural sector and offering a novel strategic approach for precision agriculture (Ghosh et al., 2023; Saberi Riseh et al., 2024; Singh et al., 2024). Research had indicated that a seed coating composed of a zinc and urea hydroxyapatite nanohybrid can more precisely deliver plant nutrients and enhance the efficiency of nutrient utilization (Abeywardana et al., 2021). Under acidic soil conditions, innovative phosphate hydroxyapatite nanofertilizers, specifically Hydroxyapatite nanoparticles (HA-NPs), were administered to sunflower crops, demonstrating a significantly faster and more efficient phosphate uptake compared to conventional phosphate and triphosphate fertilizers (Xiong et al., 2018). The design of nanocarriers represents a pivotal avenue for future research into the precise delivery of nanoparticles. The strategic development of nanocarriers that can target tissues and organs within plants and organic matter will facilitate precise control over plant absorption, decrease input requirements, and minimize energy wastage.

## 3 The Impact of nanoparticles on rhizosphere microbial communities

Nanoparticles engage in a range of physical, chemical, and biological processes, such as vulcanization, flocculation, precipitation, and adsorption, which enable them to interact with soil organic matter, plants, and root microorganisms (Chang et al., 2024). The study indicated that parameters including soil texture, pH, redox potential, organic matter content, and cation exchange capacity influence the chemical properties of nanoparticles (Ben-Moshe et al., 2013; Gao et al., 2019). When nanoparticles enter the soil system, they start to regulate the physiological, biochemical and genetic mechanisms of soil microorganisms; after entering the plant system, they were transferred to xylem tissue and then transported to other tissues to regulate the adaptability of the plant environment (Ahmed et al., 2023). In this section, we primarily explore the function and potential applications of nanoparticles in enhancing the rhizosphere soil microenvironment and fostering interactions among rhizosphere microbes.

### 3.1 Nanoparticles for enhancing the soil microenvironment in the rhizosphere

**Figure 2:**
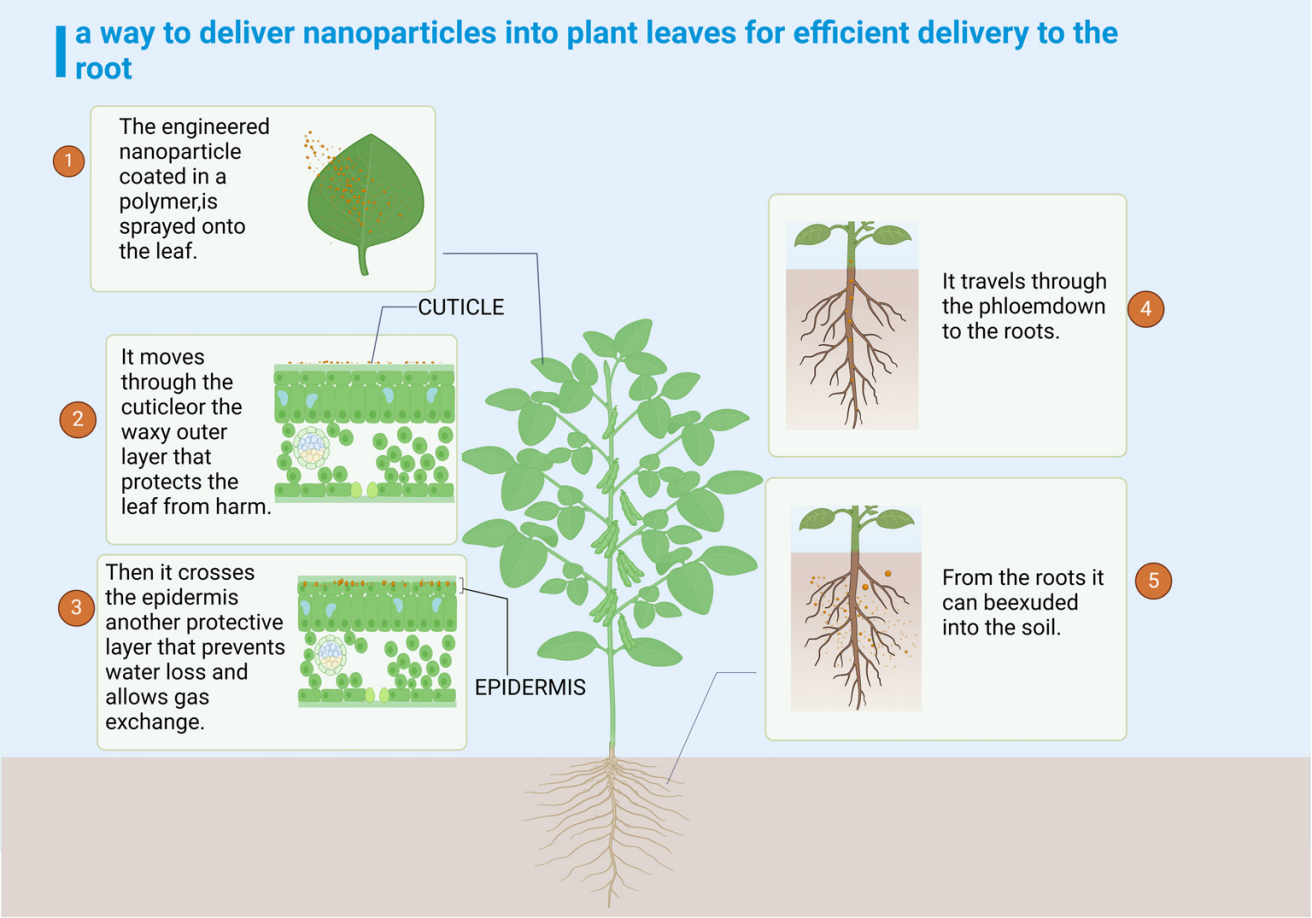
Nanoparticles are transported to the soil.

Research indicated that nanoparticles could alter soil structure by decreasing the surface energy and enhancing the water repellency of soil particles, while also increasing soil water conductivity and aggregate stability (Chen et al., 2022). These changes could impact the soil water cycle, influencing aspects such as water retention and evaporation rates. Concurrently, alterations in soil structure could also modulate the distribution of soil pH levels and nutrient elements, subsequently influencing the beneficial microbial community within the rhizosphere (Wang et al., 2020). For example, the newly developed biochar-enriched phosphorus-doped aqueous solutions, in conjunction with iron ore nanoparticles, had the capability to alter the soil pH balance, concurrently enhancing the soil’s organic matter and phosphorus concentrations (Li et al., 2022). Nanoparticles alter the structure, content, and diversity of advantageous microbial communities within plants, influencing the interaction within the plant-soil-microbial community system, and a robust soil structure further enhances the activity and nutrient cycling of soil microorganisms (Schjønning et al., 2011; Das et al., 2023; You et al., 2023; Zhang et al., 2023). Research had indicated that the application of carbon nanoparticles (CNPs) to soil could improve its water retention capacity, but also positively influence the functionality of soil microorganisms, thereby indirectly supporting plant growth (Xin et al., 2022). Concurrently, CNPs, which infiltrate plant roots via stomatal penetration or adhere to the epidermis, can stimulate soil microorganisms and enhance enzyme activities (Xin et al., 2022). This, in turn, can improve soil fertility and quality (**Figure 2**).

Nanoparticles have the potential to foster the proliferation of microbial community diversity by altering the elemental composition of the soil (Karimian and Samiei, 2023). CNPs can significantly enhance the soil’s nitrogen and phosphorus content, thereby fostering a thriving environment for microbial growth-related enzymes, which in turn promotes soil health and fertility (Zhao et al., 2021). Biologically synthesized nanoparticles exert a beneficial influence on organic carbon and microorganisms within soils used for corn cultivation, thereby accelerating plant growth (Haider et al., 2015). Innovative semi-polymer nanocomposite particles possess the capability to significantly augment the concentrations of both organic carbon and active organic carbon, thereby fostering the vigorous growth and development of plants (Zhao et al., 2019). The aforementioned studies indicate that nanoparticles have the potential to enhance soil structure, pH levels, and various other properties, which in turn can influence the interactions among plants, soil, and microbes. However, the precise manner in which these soil properties impact the interplay between these three elements warrants further investigation.

### 3.2 Nanoparticles promote the interaction between rhizosphere microorganisms and plants

The rhizosphere is regarded as the soil’s most abundant reservoir of organic matter, serving as the primary zone for microbial proliferation and activity (Reinhold-Hurek et al., 2015). The rhizosphere microbial communities play a pivotal role in various beneficial plant growth processes, including nitrogen fixation, nutrient decomposition, and the synthesis of bioactive metabolites (Bi et al., 2021; Chauhan et al., 2023). Owing to their minute particle dimensions, surface functionalization, and unique chemical properties, nanoparticles are employed to enhance plant nutrient absorption and stress tolerance (Lv et al., 2019). Presently, the majority of research into the utilization of nanoparticles is centered on the plants alone, overlooking the impact of nanoparticles on soil microorganisms and their metabolites during this process.

The interaction between rhizosphere secretions and rhizosphere microorganisms is bidirectional, indicating that the metabolites excreted by plant roots play a role in shaping rhizosphere microbial communities; conversely, rhizosphere microorganisms are intimately connected to plant growth and health (Staley et al., 2017; Hu et al., 2018; Trivedi et al., 2020). Nevertheless, a considerable amount of uncertainty persists regarding the interplay between secretions and microbes in the rhizosphere.

The interaction between nanoparticles and plants can significantly promote the generation of metabolites in plant cells and the increase of rhizosphere secretions (Francis et al., 2024). These bioactive substances, once in contact with nanoparticles, indirectly have a profound impact on their key properties such as dispersion stability, aggregation state, and solubility, which has been confirmed in relevant research (McManus et al., 2018; Cervantes-Avilés et al., 2021). Specifically, biomolecules in plant cell metabolites and rhizosphere secretions (amino acids, sugars, phenolic compounds, and other secondary metabolites), Its functional groups have high reactivity and can quickly adsorb onto the surface of nanoparticles through competitive interactions, forming a surface ecological corona (eco-corona) (Nasser and Lynch, 2016; Wheeler et al., 2021). This ecological corona not only changes the physical and chemical properties of nanoparticles, but also further affects their migration, transformation, and biological effects in the soil environment, thus forming a complex ecological interaction network (Kang et al., 2024). This ecological corona phenomenon has a significant impact on the bioavailability of nanoparticles by crops, and its effect exhibits duality. In certain specific contexts, it may have a positive impact, promoting crop uptake and utilization of nanoparticle nutrients; However, in other cases, it may also have adverse effects, interfering with the normal physiological processes of crops. When ecological corona enhances the dispersion and stability of nanoparticles in soil or rhizosphere environment, it helps crop roots to more effectively contact and uptake these nanoparticles, thereby improving bioavailability and promoting crop growth and development. The organic acids in soybean rhizosphere secretions bind to the surface of nanoparticles (CeO_2_, Mn_3_O_4_, Cu(OH)_2_, MoO_3_), greatly reducing the bioavailability and delivery efficiency of these agricultural nano chemicals (Cervantes-Avilés et al., 2021). However, if ecological corona leads to an increase in the aggregation degree or a decrease in the solubility of nanoparticles, it may hinder the effective absorption of nanoparticles by crops. For example, the rhizosphere secretion of wheat increases the solubility of CuO nanoparticles in alkaline soil, thereby enhancing their bioavailability by crops (Hortin et al., 2020). Therefore, when using nanoparticles for agricultural production, it is necessary to fully consider the impact of ecological corona phenomenon on crop bioavailability. Through scientific and reasonable nanoparticle design and application strategies, the positive effects can be maximized while minimizing potential risks, in order to achieve sustainable development of agricultural production.

Research has indicated that nanoparticles frequently influence rhizosphere microorganisms by stimulating the secretion of root metabolites. For instance, the adsorption of zinc oxide nanoparticles (ZnO NPs) and their aged counterpart, s-ZnO NPs, onto the epidermis of legume roots triggers a stress response that results in the production of numerous root metabolites, including amino acids and terpenoids (Liu et al., 2023). These metabolites could directly impact soil organic matter or activate microbial decomposition of organic carbon, thereby enhancing the release and breakdown of organic carbon within the rhizosphere (Liu et al., 2023). Selenium nanoparticles (Se NPs) enhanced organic acid biosynthesis and transport genes in plants, directly enhance malate and citric acid secretion in rice roots, and then recruit sphingomonas and Streptomyces, to enhance their interaction with rice and promote the growth of rice (Jiao et al., 2023). The application of Silica dioxide nanoparticles (SiO_2_ NPs) stimulated the synthesis, transport, and secretion of organic acids in rice roots, which provided a rich carbon source for rhizosphere microorganisms, increases the abundance of beneficial microorganisms such as *Proproteobacteria* and *Actinobacteria* in the rhizosphere by 15.2-80.5%, promotes the optimization of the bacterial community, and facilitates the absorption and growth of nitrogen in plants (Yue et al., 2023). It has been reported that compounds released by root exudates and rhizosphere microorganisms can form complexes with metal ions, thereby influencing their bioavailability to plants and microorganisms (Chen et al., 2017). Fe_3_O_4_ nanoparticles encapsulated in citrate (CA) release a higher solubility of iron and interact with root exudates, which modulate plant hormones to stimulate root elongation, thereby enhancing plant growth (Sun et al., 2023). Consequently, nanoparticles can be employed to modulate rhizosphere secretions, thereby influencing the metabolic processes and community dynamics of rhizosphere microorganisms, which in turn can facilitate the emission of plant root exudates (Steinauer et al., 2016). The studies conducted clearly indicate that nanoparticles have the capacity to modify the rhizosphere microbiome, thereby enhancing the population of beneficial microbes. To fully harness the agricultural benefits, it is imperative to gain a more profound and holistic comprehension of how nanoparticles influence the interplay between root exudates and the rhizosphere microbiome.

### 3.3 Nanoparticles suppress plant pathogenic microorganisms

**Figure 3:**
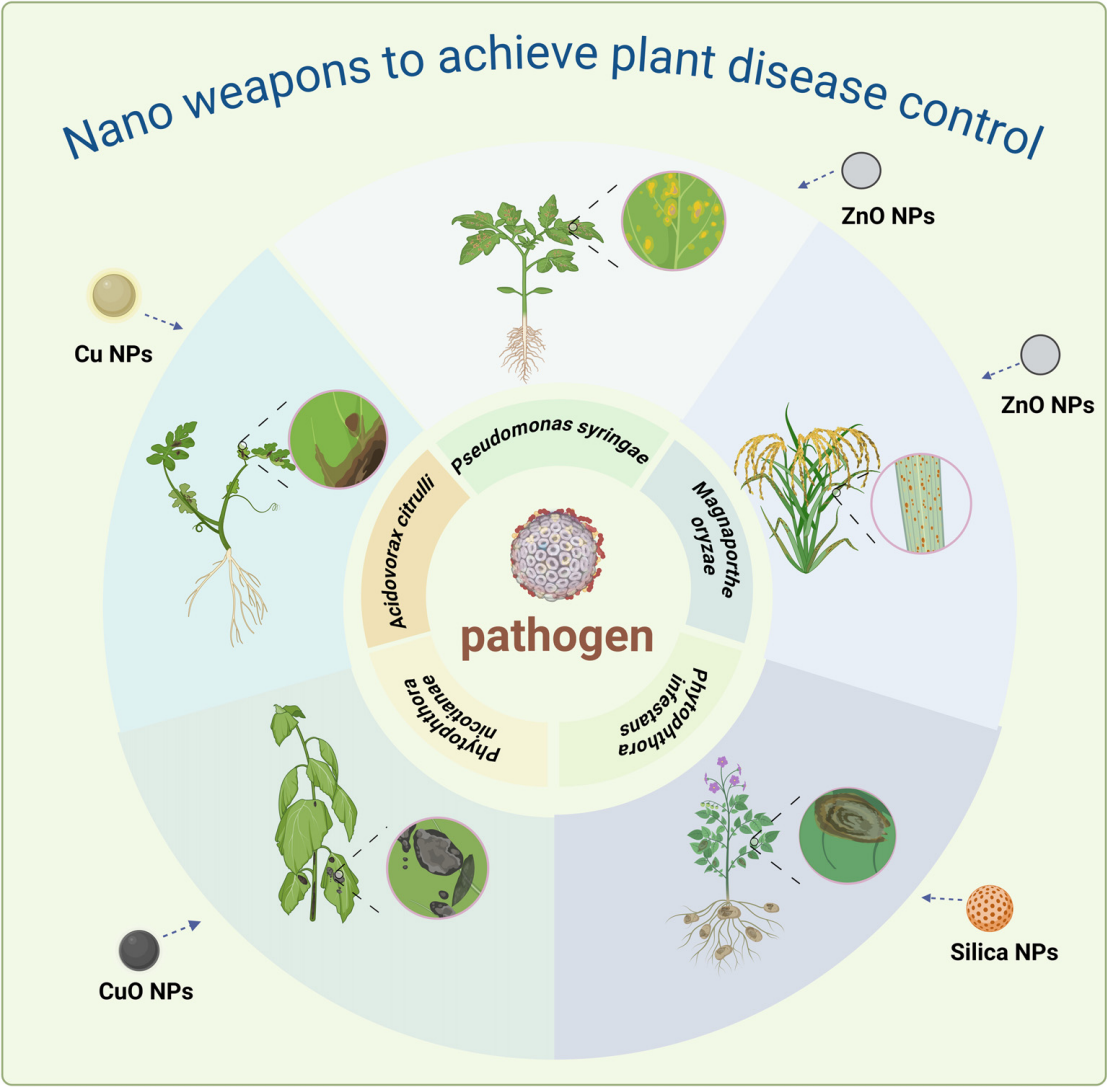
The inhibitory effects of different nanoparticles on different plant diseases.

Plant diseases can pose a formidable threat to the productivity and quality of plants. Certain nanoparticles, harnessing their exceptional cell penetration abilities and unique surface properties, demonstrated a promising potential for inhibiting a diverse array of pathogenic microorganisms, thereby ultimately fulfilling the objective of managing and controlling plant diseases effectively (Gordienko et al., 2019; Gao et al., 2023; Rahimizadeh et al., 2023; Scandolera et al., 2024). The most widely studied nanoparticles currently are those of silver, copper, zinc, silicon, etc. These particles usually possess special physical and chemical properties that enable them to interact effectively with plant pathogens (directly destroying the cell membranes of plant pathogenic bacteria, causing lysis of the pathogenic cells and a decrease in pathogenic activity) and inhibit their growth and spread, thereby effectively reducing the incidence and severity of plant diseases (Andleeb et al., 2021; Jaithon et al., 2022). **Figure 3** enumerated the application of nanoparticles in five common plant diseases.

Research has indicated that nanoparticles predominantly contribute to the management of plant diseases by disrupting the morphological structure, sporulation capacity, and adhesion behavior of pathogens (**Table 1**). Silver-associated nanoparticles currently stand out as the most effective in combating pathogenic microorganisms, with their antibacterial properties being particularly evident in organisms such as *Ustilaginoidea virens*, *Ralstonia solanacearum*, and *Xanthomonas perforans (* Pisárčik et al., 2021). The antimicrobial efficacy of these silver nanoparticles was intricately linked to their unique physicochemical characteristics, including concentration, particle size, pH, and other factors (Pisárčik et al., 2021). For example, the growth inhibition of rice false smut fungus by nanosilver was concentration-dependent, and nanosilver at a median effective concentration could significantly inhibit the sporulation and pathogenicity of the fungus. In addition, Ag NPs reduced the H3K27me3 modification mediated by UvKmt6, leading to the upregulation of genes involved in biosynthesis of oryzalide, and the decrease in H3K27me3 levels is associated with the inhibition of mycelial growth (Wen et al., 2023). In addition to combating fungal and bacterial pathogens, silver nanoparticles could also diminish the concentration of Bean Yellow Mosaic Virus (BYMV) and alleviate disease severity in broad beans (Abdelkhalek et al., 2023). Nano biotechnology has revealed that copper nanoparticles, particularly tobacco-derived copper oxide nanoparticles (CuO NPs), exhibit a pronounced antibacterial effect that is concentration-dependent. Scanning electron microscopy (SEM) and transmission electron microscopy (TEM) observations have shown that CuO NPs can disrupt the hyphal cell wall, resulting in a rough and convex surface (Chen et al., 2022). Additionally, there is evidence of significant partial collapse and bending of the hyphae (Chen et al., 2022). This phenomenon occurs because CuO NPs aggregate and adhere to the hyphal cell wall, and may even penetrate the cell membrane (Chen et al., 2022). The direct contact between nanoparticles and the hyphae leads to the accumulation of reactive oxygen species (ROS) and a corresponding increase in hyphal superoxide dismutase (SOD) enzyme activity (Chen et al., 2022). A recent study showed that copper nanoparticles (Cu NPs) also play an important role in the inhibition of bacterial fruit spot disease in watermelon, also by inducing oxidative stress and destroying cell integrity (Noman et al., 2023). **Table 1** offers a comprehensive overview of the utilization of nanoparticles as antimicrobial agents.

**Table 1:** Effect of various nanofertilizer/nanoparticles on pathogenic microbes and microbial functions.

Nanomaterials/Nanoparticles	Particle size	Dose and mode of application	Effect on Pathogenic microbes	Effect on Pathogenic microbes functions/Effect on plant	Reference
Metal Silver (Ag) related	2 nm	2.16 μg/mL, direct application to soil	Both the surface and intracellular organelles of *Ustilaginoidea virens* were disrupted, and affect mycelial growth, conidiation, and virulence of *U*. *virens*	Affects several energy utilization and metabolic processes in *Ustilaginoidea virens*	(Wen et al., 2023)
	21/29 ± 5 nm	400 μg/mL, direct application to soil	*Ralstonia solanacearum* envelope is damaged, the cells bulge and form small pits.	The cellular metabolic activity and surface adhering ability of *R. solanacearum* were completely lost	(Haroon et al., 2019)
	18 nm	100 ppm, direct application to soil	Led to cell deformation and loss of the rod-shaped structure of the *Xanthomonas perforans*	Significantly reduced the severity of bacterial spot disease	(Ocsoy et al., 2013)
	5-35 nm	20 µg/mL, direct application to soil	*Ralstonia solanacearum* cell wall and plasma membrane rupture, as well as nucleic acid material leakage	Inhibit the growth, community and swimming movement of *Ralstonia solanacearum*	(Abd Alamer et al., 2022)
Metal copper (Cu) related	10-100 nm	100 mg/L, direct application to soil	Cell wall damage of, such as rough and convex cell envelope, accompanied by obvious local collapse and distortion	Activated a series of defense enzyme activities in tobacco	(Chen et al., 2022)
	29.11-78.56 nm	100 µg/mL, foliar spray	Induced oxidative stress, biofilm inhibition and cell integrity destruction in *Acidovorax citrulli*	Regulate host’s active immune response to inhibit watermelon bacterial fruit blotch	(Noman et al., 2023)
	200-500 nm	0.5 mg/mL, direct application to soil	Damage the cell membrane of *Fusarium oxysporum f.* sp. *lycopersici*, altering the permeability of the cell membrane, leading to the disintegration of the cell membrane, and eventually to cell death	Effectively treats Fusarium wilt while promoting the growth of tomato plants	(Lopez-Lima et al., 2021)
Metal iron (Fe) related	86 nm	250 μg/mL, foliar spray	*Xanthomonas oryzae pv. oryzae* cell membrane destruction, ROS formation, DNA damage, protein and enzyme degeneration, and leakage of intracellular contents ultimately lead to cell death	Maintaining ionic homeostasis,and improving the photosynthetic profile	(Ahmed et al., 2022)
Metal zinc (Zn) related	30 nm	200 mg/L, direct application to soil or foliar spray	Direct antifungal activity against *M. oryzae* by inhibiting its conidiation and appressorium formation	Fight against blast disease,and enhance the tolerance of rice seedlings to abiotic stress	(Qiu et al., 2023)
	0.323 nm	100 μg/mL, foliar spray	Destroyed *Pseudomonas syringae* pv. tomato DC3000 membrane and induces deformation of the contents of the cytoplasm, leading eventually to cell death	Protect tomato against the bacterial speck pathogen,and promote plant growth	(Elsharkawy et al., 2020)
Silica (Si) related	5-15 nm	0.2 g/L, foliar spray	*Fusarium oxysporum* f. sp. *lycopersici* and *Alternaria solani* showed disturbed and fragmented mycelium	Enhance plant growth, photosynthetic pigments and reduce the disease indices	(Parveen and Siddiqui, 2022)
	80-100 nm	300 mg/L, foliar spray	*Phytophthora infestans* structurally distorted with terminal deformity and local shrinkage	Prevented the appearance of small brown spots and aerial mycelium on the potato tubers	(Chen et al., 2023)
Chitosan nanoparticles	NA	1g/L,inoculated into the ginger rhizomes wound	Inhibited the mycelial growth and spore germination of *Fusarium solani*	The antioxidant defense system of ginger at a high level in response to hence reduced disease indices	(Zhang et al., 2024)

#NA, Not available.

In conclusion, the antimicrobial properties of nanoparticles are derived from their unique physical structure and chemical reactivity. Nonetheless, despite their demonstrated efficacy in inhibiting pathogenic microorganisms, practical applications must take into account their stability, biocompatibility, environmental impact, and cost-effectiveness.

### 3.4 Nanoparticles boost the efficacy of probiotic microorganisms

**Figure 4:**
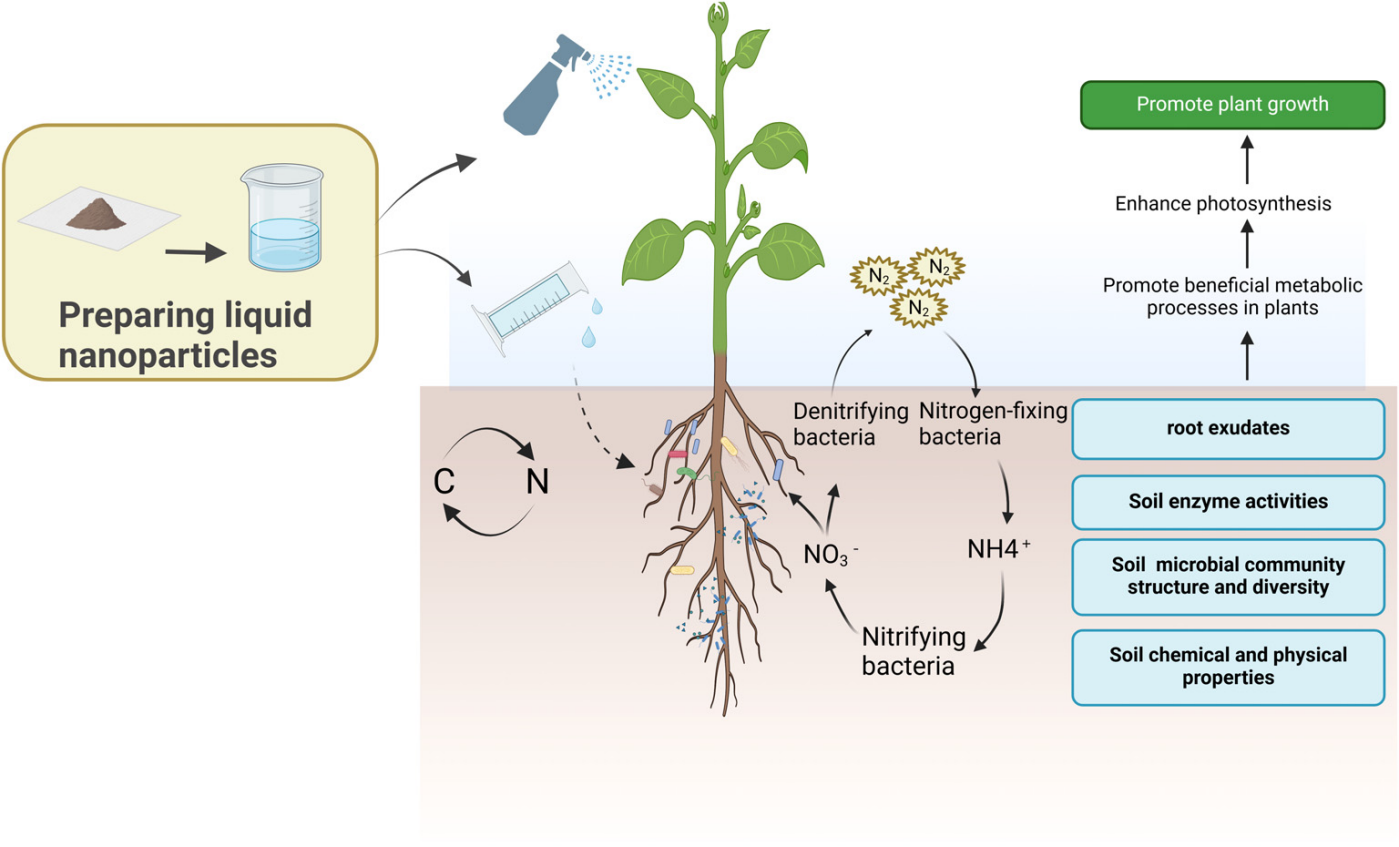
Nanoparticles enhance the activity of beneficial microbes within the rhizosphere, modulate soil metabolic processes, and subsequently foster plant growth and development.

Numerous nanoparticles have the potential to improve nutrient transport and soil fertility by modulating the function, species diversity, or population size of the microbial community within the rhizosphere (Wei et al., 2020). Foliar application or direct application of nanoparticle solutions could influence the function of rhizosphere microbial communities in plant root nutrient uptake, nitrogen regulation, and the regulation of related enzyme activities, thereby impacting plant growth and development (**Figure 4**) (Wu et al., 2023).

Throughout the phase of accelerated plant growth, the high nutrient demand entices a larger concentration of rhizosphere microorganisms, which play a crucial role in the plants’ nutrient absorption (Chaparro et al., 2014). Simultaneously, the interplay between nanoparticles and rhizosphere microorganisms could enhance plant growth and improve soil health (Tripathi et al., 2024). The research indicated that in the developmental phase of rice seedlings, selenium nanoparticles (Se NPs) enhance the abundance of sphingomonas and various other bacterial species, while also encouraging the secretion of root exudates (Jiao et al., 2023). These combined influenced synergistically modulate nutrient uptake and foster the growth of rice plants (Jiao et al., 2023).In *Brassica chinensis L.*, there exists a comparable regulatory function throughout the growth process (Wang et al., 2022). The symbiotic relationship between rhizosphere microorganisms and plants enhanced the bioavailability of nutrients within the rhizosphere soil, thereby augmenting the plants’ nutrient uptake capabilities (Xu et al., 2023). The application of zinc oxide quantum dots (ZnO QDs) during the growth phase of pumpkins aids in enhancing beneficial microorganisms within the endophytic and rhizosphere environments, thus promoting nutrient uptake and plant growth (Xu et al., 2023). It has been shown that the 50 mg/kg of Fe_7_(PO_4_)_6_ nanoparticle treatment of tomato will increase the relative abundance of beneficial microorganisms associated with nutrient accumulation, which will accelerate nutrient accumulation (Jiao et al., 2024).

Numerous studies have demonstrated that the availability of soil nitrogen and the uptake of nitrogen by plants play a crucial role in determining crop yield. Concurrently, the absorption, excretion, and transformation of soil nitrogen are largely contingent upon the interactions within the rhizosphere microbial communities (Yang et al., 2019; Yu et al., 2019). The effective and sustainable supply of nitrogen in the rhizosphere soil mainly depends on specific rhizosphere microbial communities that convert inert nitrogen into nitrogen compounds. Therefore, nitrogen transformation driven by rhizosphere microbial communities was a significant determinant of plant nitrogen uptake (Moreau et al., 2019). For example, the application of FeNPs to alfalfa increased the diversity of the rhizosphere microbial community, further enhancing the nitrogen-fixing ability of the roots (Zhang et al., 2024). Silver nanoparticles (AgNPs) enhanced the population of rhizosphere bacteria, including *Saccharimonadia* (also known as Plant Growth Promoting Rhizobacteria, or PGPR) (Sellstedt et al., 2013; Francioli et al., 2021). Certain strains of these bacteria facilitate atmospheric nitrogen fixation, while others contribute to plant growth and aid in nutrient transformation (Sellstedt et al., 2013; Francioli et al., 2021). **Table 2** offers a comprehensive overview of the enhancement of diverse nanoparticles on various beneficial microbes within the rhizosphere, along with their affirmative impacts on plant growth.

**Table 2:** Effect of various nanoparticles on rhizospheric microorganisms and microbial functions.

Nanoparticles	Particle size	Name of the plant	Dose and mode of application	Effect on rhizosphere microorganisms	Effect on rhizosphere microorganisms functions	Effect on plant	Reference
Metal Silver (Ag) related	20 nm	Rice	1 mg/kg, direct application to soil	Increase the number of putative beneficial soil bacteria, *eg*, *Frankiales*, *Rhizobiales*, *Chitinophagales*, and *Saccharimonadia*	Improved biogeochemical cycling of nitrogen, carbon etc.	Promote tillering and increase grain yield	(Yan et al., 2022)
	30-60 nm	*Triticum aestivum*	10 ppm, direct application to soil	Increase the number of putative beneficial soil bacteria, such as *Pseudomonas* spp. *and Arthrobacter* spp.	Enhanced nitrogen fixation	Increased growth and yield of plant	(Przemieniecki et al., 2024)
Metal copper (Cu) related	28 ± 14 nm	*Triticum aestivum*	0.5 mg/kg, direct application to soil	The abundance of *Sphingobacterium*, *Pseudomonas*and other bacteria communities increased	Improved nitrogen fixation and reduced denitrification process	Enhance plant photosynthesis	(Guan et al., 2020)
Metal iron (Fe) related	83 nm	Tomato	50 mg/kg, direct application to soil	Enhance the abundance of beneficial genera in the rhizosphere, particularly *Nitrospira*, *Sphingomonas*, *Massilia*, *Bryobacter*, *Chithonibacter*, *RB41* and *Rubellimicrobium*	Enhanced nitrogen fixation	Significantly enhanced nutrient uptake, improved flowering development, and ultimately increased nutritional quality and tomato yield	(Jiao et al., 2024)
	4–10 nm	*Glycine max*	30 mg/L, foliar spray	Enhanced rhizobial activity	Enhanced nitrogen fixation	Improved the soybean yield and promoted the nutritional quality	(Cao et al., 2022)
	NA	*Medicago sativa* L.	10 mg/L, seed soaking or foliar spray	Increase the number of putative beneficial soil bacteria, such as *Proteobacteria, Firmicutes, Actinobacteria, and Bacteroidetes*	Improved nitrogen fixation and reduced denitrification process	Enhance photosynthesis and promote growth	(Zhang et al., 2024)
Metal zinc (Zn) related	4.06 nm	*Cucurbita moschata* Duch.	0.61 mmol/L, foliar spray	Increased abundance of *Steroidobacter* and *Paenibacillus*	Improve nitrogen metabolism	Promote plant growth, nutrient absorption, and tolerance to stress	(Xu et al., 2023)
	22-28 nm	*Medicago truncatula*	100 mg/kg, direct application to soil	Increase the number of putative beneficial soil bacteria,such as *Haliangium*, *norank_f:BIrii41*, *Gaiella*, *norank_f:Gemmatimonadaceae* and *Lysobacter*	Improve the underground carbon cycle and the breakdown of cellulose	Promote plant growth	(Liu et al., 2023)
	18 nm	*Sorghum bicolor* var. 251	5 mg/kg, direct application to soil	Microbial quantity and activity were enhanced	Improve nitrogen metabolism	Accelerate plant growth and increase yield	(Christian et al., 2019)
Selenium (Se) related	3–18 nm	*Oryza sativa* L.	0.1 mg/kg, direct application to soil	Increase the number of putative beneficial soil bacteria, such as *Streptomyces* and *Sphingomonas*	Improves the adhesion of beneficial bacteria to roots	Promote plant growth	(Jiao et al., 2023)
Silica (Si) related	20 nm	*Brassica chinensis* L.	Each dose is 1 mg,foliar spray	Increased abundance of *Paenibacillus*, *Rhodobacteraceae*, *Chaetomium* while abundance of silicate *Rhodoplane* was reduced	Improved biogeochemical cycling of nitrogen, carbon	Promote root growth	(Tian et al., 2020)
	8 nm	*O. sativa* L.	50 mg/kg, direct application to soil	Increase the number of putative beneficial soil bacteria,such as *Proteobacteria, Actinobacteriota* etc.	Improved nitrogen fixation and reduced denitrification process	Promote the growth and yield of rice through enhancement of tillering	(Yue et al., 2023)

#NA, Not available.

Although numerous studies have extensively reported the positive effects of nanoparticles on plant and rhizosphere microbial community diversity, there are still some studies suggesting that the effects of nanoparticles on plants and microorganisms may exhibit dose-dependent effects, especially under high concentration conditions, where they may have inhibitory effects on plant growth and some rhizosphere microbial communities (Saghaï et al., 2022; Ren et al., 2024). For example, in plant growth experiments, low concentrations of ZnO nanoparticles promoted the growth of *Vigna radiata* and *Cicer arietinum* seedlings. Treatment concentrations of nanoparticles higher than 20 ppm and 1 ppm, respectively, would inhibit plant growth (Mahajan et al., 2011). At the same time, 1.2 mM low concentration ZnO nanoparticles can also promote the germination and metabolic activity of *Solanum lycopersicum*, while the germination and metabolic activity of plants above this concentration are inhibited (Singh et al., 2016). High concentrations of nanoparticles not only affect seed germination and plant growth rate, but also affect the level of edible nutrients. Low concentrations of TiO_2_ nanoparticles (50 mg/L) can increase the level of nutrients in *Coriandrum sativum* L., while high concentrations can reduce the decrease in edible nutrient content and inhibit growth. In terms of microbial activity, it usually follows a dose-dependent pattern, which is also related to the type of nanoparticles (Yang et al., 2021; Lin et al., 2022). Low dose nanoparticles may have beneficial effects on soil rhizosphere microorganisms by promoting metabolism and energy conversion. For example, treating *Medicago truncatula* with 5 mg/kg of Ag and 50 mg/kg of Zn, and Ti nanoparticles significantly increased the types and total amount of soil rhizosphere microorganisms (Chen et al., 2017). Low dose (10 mg/kg) ZnO nanoparticles promote the proliferation of Cyanobacteria in the rhizosphere of *Lactuca sativa* L., but 100 mg/kg ZnO has no significant effect on this colony under the same treatment (Xu et al., 2018). Therefore, excessive application of nanoparticles is likely to disrupt the balance of soil ecosystems. In order to fully utilize the benefits of nanoparticles and reduce their potential risks, it is necessary to conduct in-depth research on the interaction mechanism between nanoparticles and microorganisms, and explore reasonable usage methods and dosages. At the same time, it is necessary to strengthen the environmental risk assessment and supervision of nanoparticles to ensure their safe application.

There are also specific effects between microbes and soil enzymes (Philippot et al., 2024). A variety of soil enzymes play a crucial role in biochemical processes, facilitating the breakdown of complex organic matter and the mobilization of nutrients, while also influencing the functionality of certain microorganisms (Wen et al., 2024). In soils rich in organic matter, a greater abundance of microorganisms correlates with heightened enzyme activity, thereby enhancing the interaction between these two components (Donald et al., 2018). The utilization of ZnO nanoparticle to mung bean plants increased the diversity of soil phosphatase and phytase activities and microorganisms, and improved the level of phosphorus acquisition, while also enhancing soil health and nutrient cycle (Raliya et al., 2016). Studies have shown that the absorption of nitrogen, phosphorus and potassium in 200 mg/kg CNPs was significantly increased by 185%, 30.4% and 193%, respectively (Xin et al., 2022). The increase of plant roots was higher than that of branches, and carbon nanoparticles enhanced the activity of most soil enzymes, thus affecting the soil microbial function, thus indirectly regulating plant growth (Xin et al., 2022). These studies enable us to gain deeper insights into the ways in which nanoparticles can enhance plant nutrient absorption. However, the mechanisms underlying the complex interactions among nanoparticles, plant roots, and soil have yet to be fully investigated.

## 4 Conclusion

The above research results indicate that nanoparticles play an important role in promoting plant nutrient absorption and rhizosphere microbial communities. There is a close connection and synergistic effect between the two, jointly affecting the growth and development of plants, as well as the stability and sustainability of ecosystems. Nanoparticles can promote plant nutrient absorption by loading nutrients and accurately deliver nutrients to different parts of the plant, thereby improving nutrient utilization efficiency. Concurrently, nanoparticles also play a pivotal role in modulating rhizosphere microorganisms. They not only foster plant growth and enhance yield by combating pathogenic microorganisms that are detrimental to plants, but also alter the composition of the rhizosphere microbial community by modifying soil physical and chemical properties and root exudates, ultimately influencing the growth and development of plants.

Nanoparticles enhance plant growth and development by facilitating nutrient uptake, modulating rhizosphere microorganisms, and enhancing soil physiochemical properties, among other benefits, with a focus on their positive effects on plants. Further research into the interactions between roots and microbes in the rhizosphere influenced by nanoparticles will elucidate the mechanisms behind plant phenotypic alterations induced by nanoparticles. This will also shed light on the manipulation of the plant rhizosphere microbiome by nanoparticles, paving the way for more sustainable agricultural practices.

Various types of nanoparticles have played important roles in plant growth, but metal nanoparticles have the widest application prospects in agricultural production. They can serve as pesticide carriers, improve pesticide utilization, and reduce environmental pollution; At the same time, it can also serve as a trace element or plant growth regulator, promoting plant growth and improving yield and quality. In addition, metal nanoparticles have shown great potential in environmental monitoring and biosensing, providing timely and accurate environmental data for agricultural production. Although there are still some challenges in practical applications, such as assessing environmental effects and biosafety, as well as optimizing production costs, with the continuous development and improvement of nanotechnology, the application of metal nanoparticles in agriculture will become more extensive and in-depth, making important contributions to agricultural production and sustainable development.

Nanoparticles have the potential to directly influence plant growth and indirectly affect the surrounding ecological environment, thereby ensuring the sustainable development of agriculture and fostering the green revolution. Moving forward, it is imperative to conduct additional research to uncover the effects of nanoparticles (NPs) on higher plants, particularly crops and vegetables, when applied to soils with varying properties. This should include an exploration of the molecular mechanisms of NPs uptake, transformation, and its impact on growth parameters, as well as the interaction mechanisms between NPs and rhizosphere microbes. Concurrently, greater focus should be placed on the interplay among rhizosphere microbial communities, soil, NPs, and plants. Further studies are necessary to ascertain the beneficial effects of NPs. Moreover, long-term investigations into cereal crops and other key crops are essential to establish correlations between NP dosage, soil type, and ecological impacts. Such research is crucial for reducing reliance on chemical pesticides and fertilizers and for securing the future sustainable development of agriculture.

In summary, this review encapsulates the outcomes of nanoparticles in enhancing plant growth and modulating the rhizosphere microbiome via various mechanisms, offering a foundation for the synergistic integration of future research endeavors in the realms of nanoscience, sustainable agriculture, and environmental science.
